# Supplementary material for: Structural basis for lysophosphatidylserine recognition by GPR34
Source: Nat Commun. 2024 Feb 7;15:902. doi: 10.1038/s41467-024-45046-z (PMC10850092; doi:10.1038/s41467-024-45046-z)
Supplement: Supplementary file 1 — Supplementary Information [file 41467_2024_45046_MOESM1_ESM.pdf]

Supplementary Information

Structural basis for lysophosphatidylserine recognition by GPR34

Tamaki Izume, Ryo Kawahara, Akiharu Uwamizu, Luying Chen, Shun Yaginuma, Jumpei Omi, Hiroki Kawana, Fumiya K. Sano, Tatsuki Tanaka, Kazuhiro Kobayashi[1], Hiroyuki H. Okamoto, Yoshiaki Kise, Tomohiko Ohwada, Junken Aoki, Wataru Shihoya, Osamu Nureki

Supplementary Table 1. Cryo-EM data collection, refinement, and validation statistics.

	S3E-LysoPS-GPR34-G_i	S3E-LysoPS-GPR34-G_i (Receptor focused)	M1-GPR34-G_i	M1-GPR34-G_i (Receptor focused)
EMDB	EMD-38215	EMD-38217	EMD-38218	EMD-38219
PDB	8XBE	8XBG	8XBH	8XBI
**Data collection**				
Microscope	Titan Krios (Thermo Fisher Scientific)			
Voltage (keV)	300			
Electron exposure (e–/Å^2)	50			
Detector	Gatan K3 summit camera (Gatan)			
Magnification	× 105,000			
Defocus range (μm)	-0.8 – -1.6			
Pixel size (Å/pix)	0.83			
Number of movies	2,358		2,674	
Symmetry	C1			
Picked particles	2,012,061		1,398,276	
Final particles	79,925		236,096	
Map resolution (Å)	3.23	3.43	2.83	3.06
FSC threshold	0.143			
**Model refinement**				
Atoms	8,809	2,561	8,912	2,576
R.m.s. deviations for ideal				
Bond lengths (Å)	0.0032	0.0155	0.003	0.0129
Bond angles (°)	0.63	1.54	0.67	1.290
**Validation**				
Clashscore	9.18	9.03	10.46	7.34
Rotamers (%)	0	0.34	0	0
Ramachandran plot				
Favored (%)	94.83	95.21	96.07	98.31
Allowed (%)	5.08	4.79	3.84	1.69
Outlier (%)	0.09	0	0.09	0

Supplementary Table 2. Mutagenesis analysis of GPR34.

Parameters obtained from the TGFα shedding assay. The values of pEC_{50}, EC_{50}, E_{max}, and log_{10} relative intrinsic activity (RAi) toward two agonists are shown with the log_{10} mean fluorescence intensity (MFI), indicating the cell surface expression level of each receptor. Source data are provided as a Source Data file.

Construct		Ligand response										Surface expression	
		S3E-LysoPS					M1						
Mutation	BW position	pEC_{50}	EC_{50}	E_{max}	LogRAi	*n* =	pEC_{50}	EC_{50}	E_{max}	LogRAi	*n* =	LogMFI	*n* =
WT		8.02 ± 0.04	9.6 nM	10 ± 1%	0	8	7.86 ± 0.01	14 nM	13 ± 1.2%	0	8	2.69 ± 0.05	10
R110A	2.6	7.88 ± 0.12	13 nM	7.7 ± 1.7%	-0.25 ± 0.11	5	7.68 ± 0.13	21 nM	7 ± 1.5%	-0.49 ± 0.13	5	2.49 ± 0.03	5
K128A	3.26	8.00 ± 0.09	10 nM	7.4 ± 1.8%	-0.16 ± 0.1	5	6.35 ± 0.19	450 nM	13 ± 2%	-1.54 ± 0.15	5	2.59 ± 0.06	5
Y135A	3.33	< 5.5	> 3 μM	< 2%	< -3	5	< 5.5	> 3 μM	< 2%	< -3	5	2.61 ± 0.07	5
Y135F	3.33	7.74 ± 0.04	18 nM	8.5 ± 1.9%	-0.36 ± 0.06	5	7.36 ± 0.05	43 nM	7.3 ± 1.6%	-0.79 ± 0.05	5	2.55 ± 0.05	5
M136A	3.34	8.08 ± 0.08	8.2 nM	6.1 ± 1.2%	-0.15 ± 0.08	5	7.71 ± 0.03	20 nM	10 ± 1.5%	-0.27 ± 0.03	5	2.61 ± 0.06	5
M189A	4.6	7.65 ± 0.08	22 nM	9.1 ± 1.5%	-0.40 ± 0.08	5	8.23 ± 0.04	5.9 nM	14 ± 1.7%	0.40 ± 0.05	5	2.63 ± 0.07	5
F205A	ECL2	< 5.5	> 3 μM	< 2%	< -3	5	< 5.5	> 3 μM	< 2%	< -3	5	2.47 ± 0.06	5
H206A	ECL2	7.47 ± 0.12	34 nM	17 ± 3.5%	-0.34 ± 0.13	5	6.83 ± 0.09	150 nM	25 ± 3.8%	-0.78 ± 0.08	5	2.60 ± 0.07	5
Y207A	ECL2	6.07 ± 0.04	850 nM	15 ± 3.3%	-1.77 ± 0.04	5	6.21 ± 0.03	610 nM	14 ± 3%	-1.65 ± 0.05	5	2.66 ± 0.09	5
R208A	ECL2	6.68 ± 0.26	210 nM	12 ± 3.2%	-1.29 ± 0.21	5	6.71 ± 0.10	190 nM	14 ± 2.2%	-1.15 ± 0.08	5	2.57 ± 0.05	5
K210A	ECL2	8.30 ± 0.11	5 nM	15 ± 2%	0.45 ± 0.11	5	7.29 ± 0.06	51 nM	18 ± 3.3%	-0.45 ± 0.07	5	2.64 ± 0.04	5
F219A	5.39	7.52 ± 0.08	30 nM	16 ± 2.6%	-0.28 ± 0.07	5	8.36 ± 0.04	4.3 nM	22 ± 2.8%	0.71 ± 0.05	5	2.69 ± 0.07	5
N220A	5.4	7.71 ± 0.10	19 nM	14 ± 2.1%	-0.15 ± 0.09	5	6.87 ± 0.09	140 nM	18 ± 3.9%	-0.89 ± 0.08	5	2.55 ± 0.04	5
L223A	5.43	7.41 ± 0.27	39 nM	14 ± 2.7%	-0.47 ± 0.23	5	6.84 ± 0.06	140 nM	16 ± 2.8%	-0.96 ± 0.07	5	2.52 ± 0.05	5
R286A	6.55	< 5.5	> 3 μM	< 2%	< -3	5	< 5.5	> 3 μM	< 2%	< -3	5	2.55 ± 0.04	5
Y289A	6.58	< 5.5	> 3 μM	< 2%	< -3	5	< 5.5	> 3 μM	< 2%	< -3	5	2.50 ± 0.03	5
N309A	7.35	6.87 ± 0.09	130 nM	12 ± 2.9%	-1.09 ± 0.09	5	7.06 ± 0.05	87 nM	11 ± 2.4%	-0.91 ± 0.07	5	2.48 ± 0.04	5
E310A	7.36	6.50 ± 0.11	320 nM	9.1 ± 2.6%	-1.58 ± 0.10	5	6.70 ± 0.07	200 nM	7.6 ± 1.8%	-1.44 ± 0.10	5	2.45 ± 0.04	5

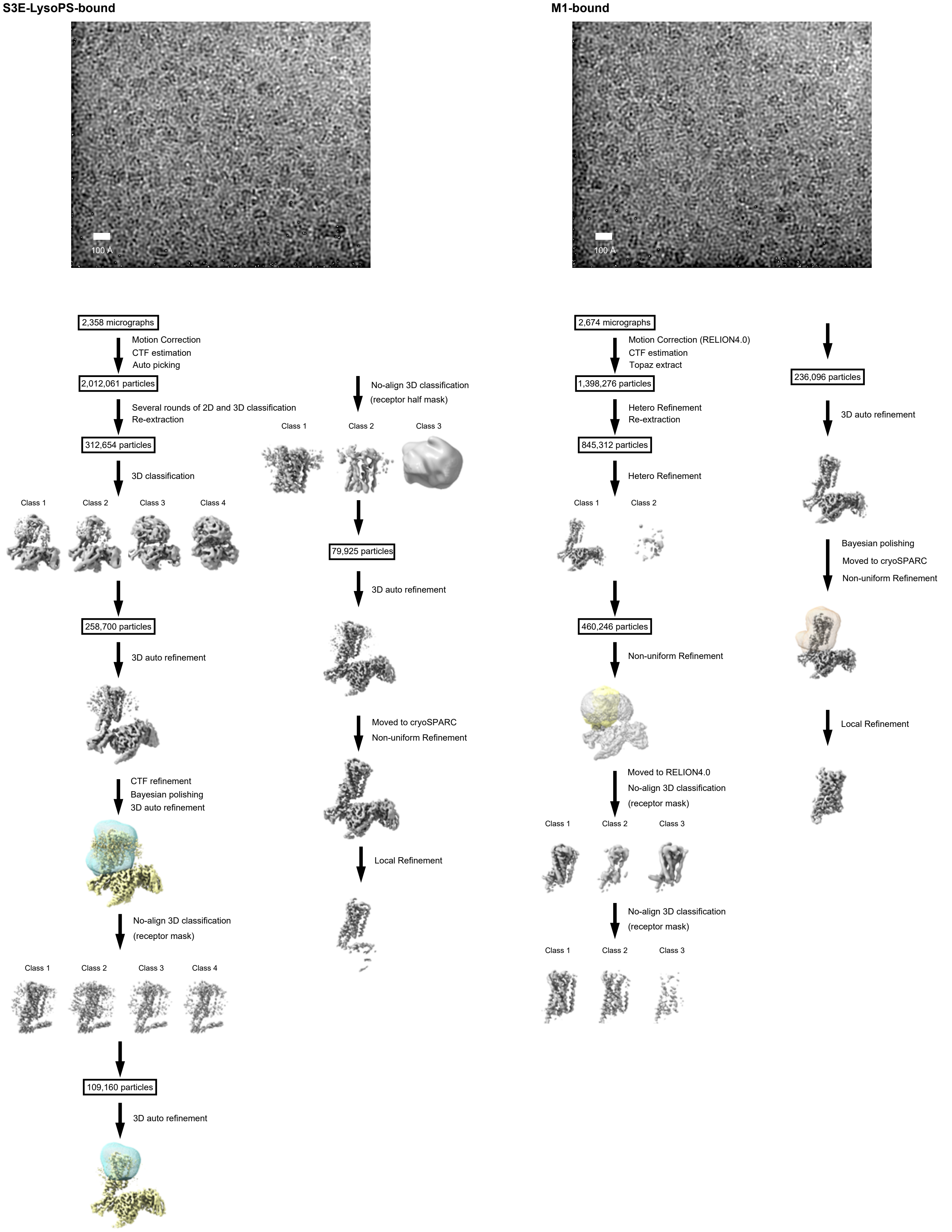

Supplementary Fig. 1 Cryo-EM workflows of the GPR34-G$_i$ complexes.

Flow chart of the cryo-EM data processing for the GPR34-G$_i$ complexes bound to S3E-LysoPS and M1, including particle projection selection, classification, and 3D density map reconstruction. Details are provided in the Methods section.

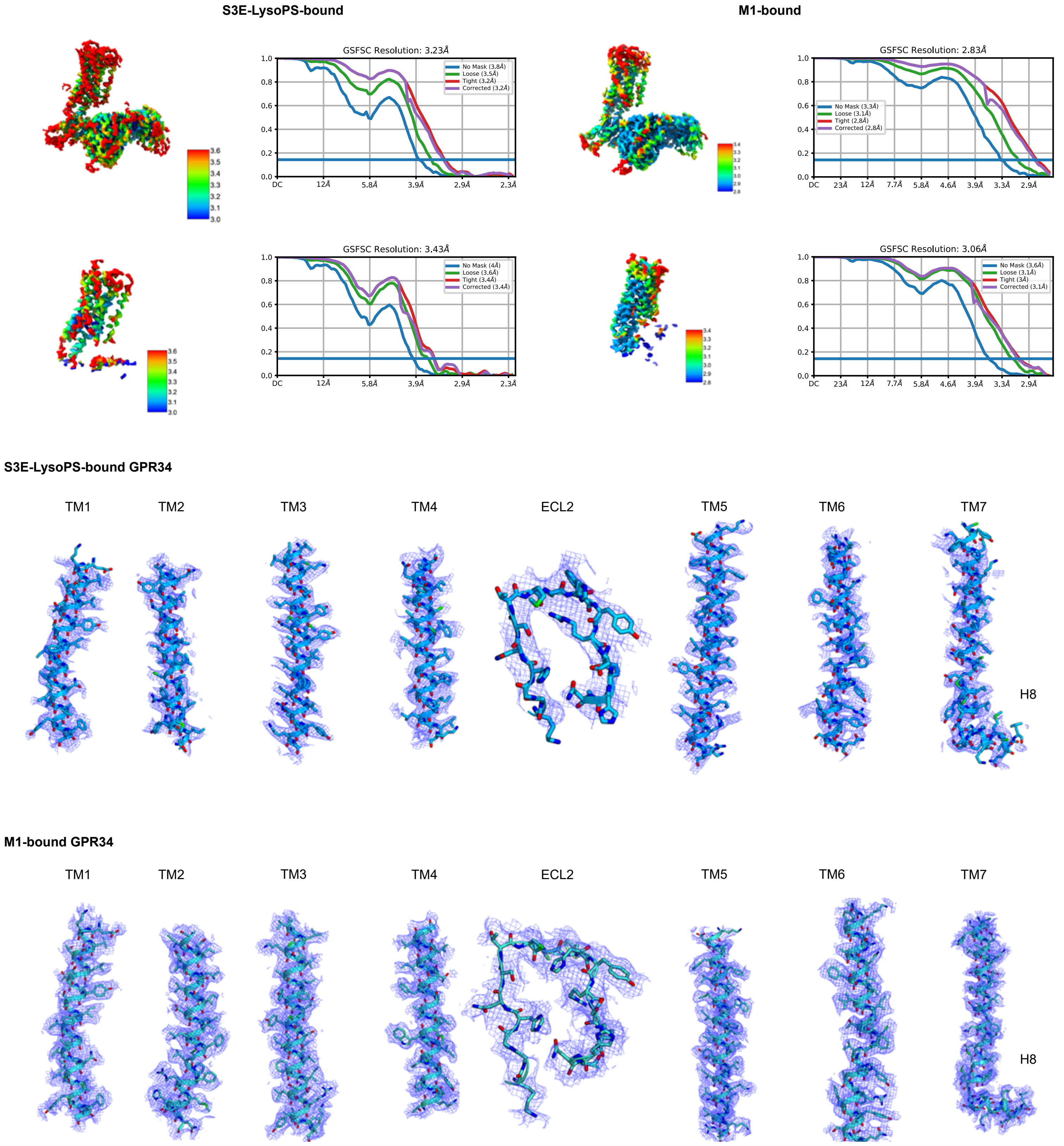

Supplementary Fig. 2 Maps and model quality.

Local resolution maps, FSC curves, and cryo-EM density maps of the GPR34-G_i complexes bound to S3E-LysoPS and M1.

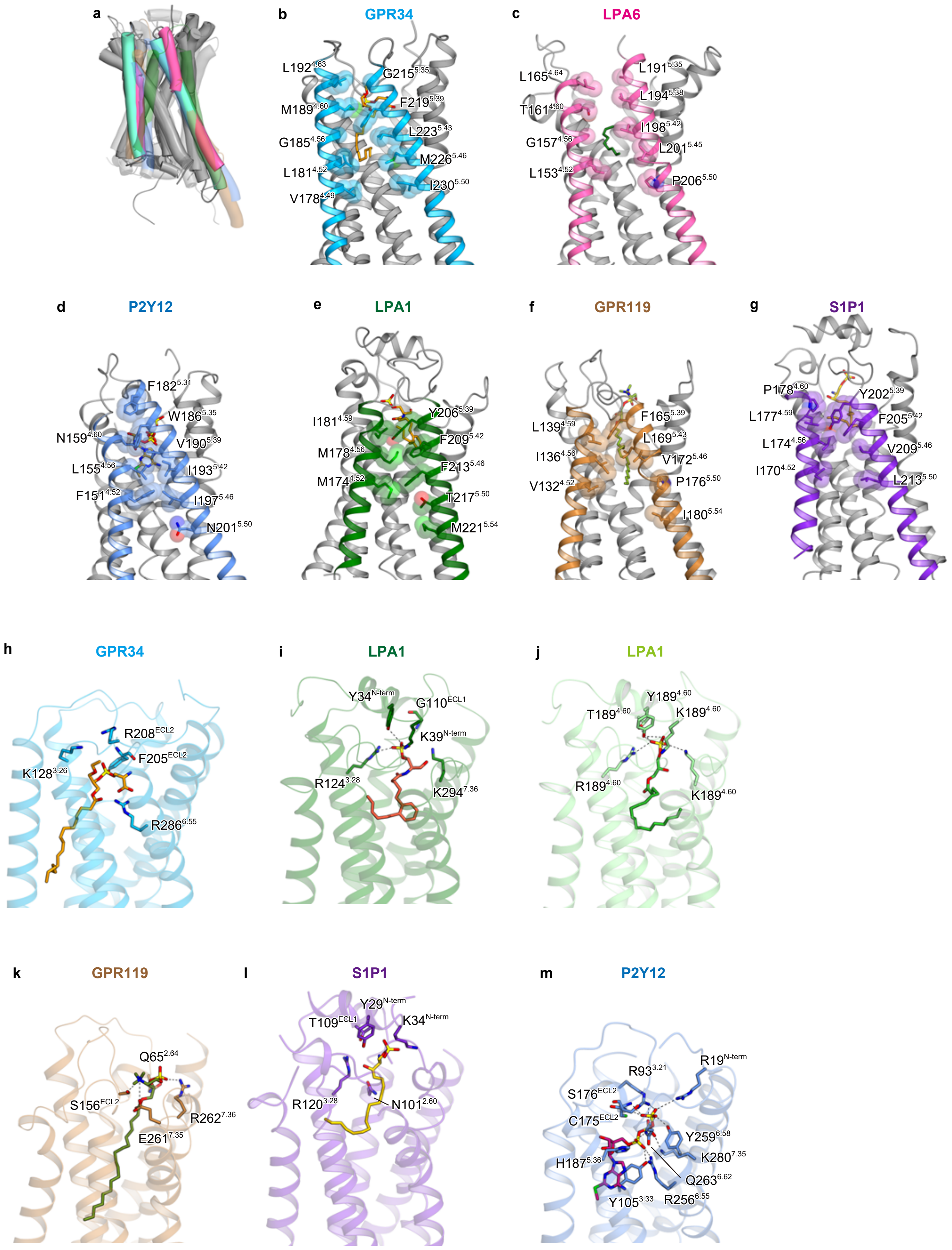

Supplementary Fig. 3 Structural comparison of GPR34 with other GPCRs.

(a) Superimposition of the structures of GPR34, LPA$_6$, LPA$_1$, GPR119, and S1P$_1$.

(b-g) Interactions between the extracellular halves of TM4 and TM5 in respective receptors.

(h-m) Comparison of the agonist binding modes in respective receptors.

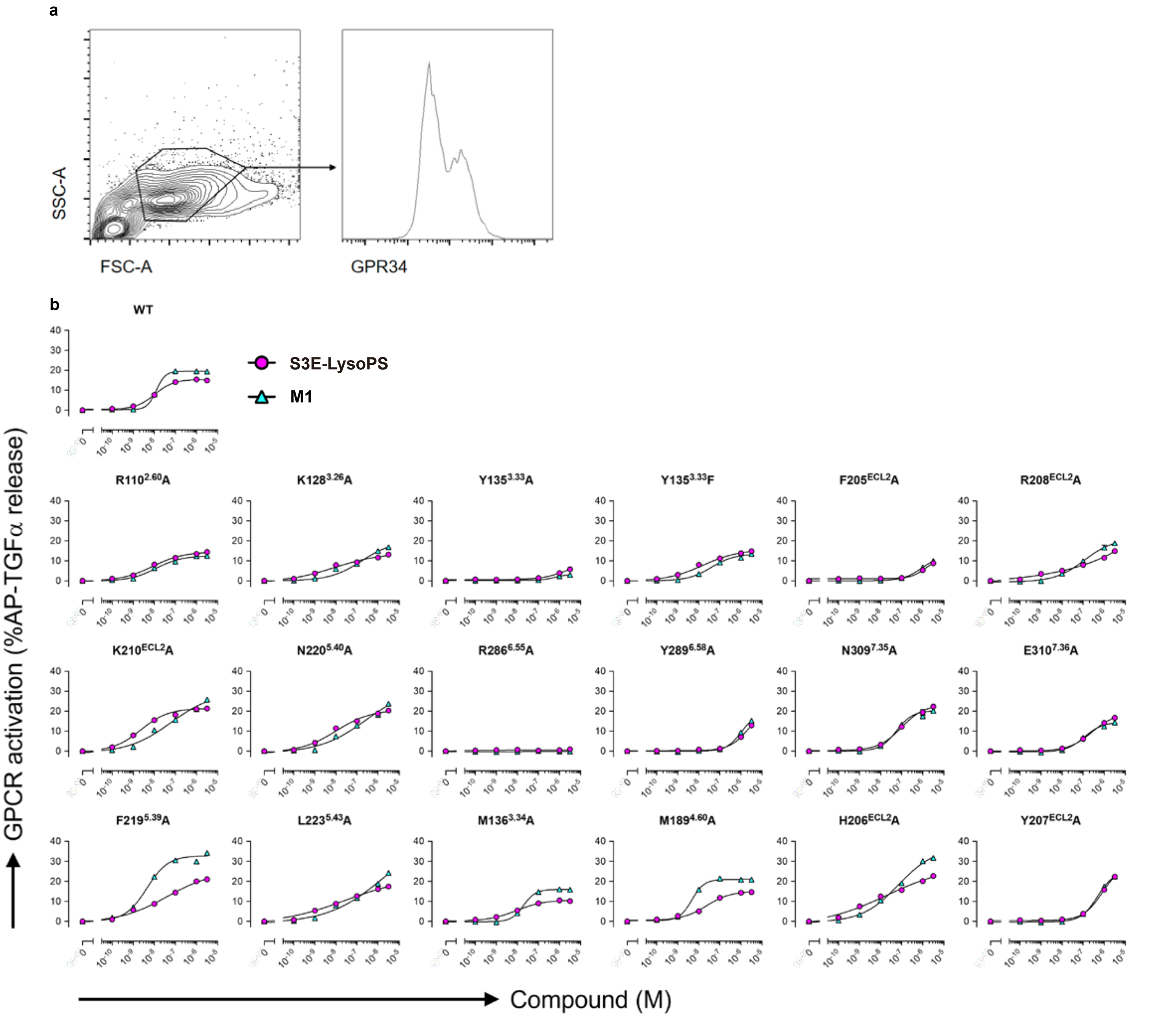

Supplementary Fig. 4 Mutational analyses using the TGFα shedding assay.

(a) Gating strategy used to evaluate cell-surface expression of GPR34 proteins. Geometric MFI values calculated based on GPR34 histogram (right panel) are presented on Supplementary Table 2. **(b)** The responses of wild-type GPR34 and 18 mutants of GPR34 toward S3E-LysoPS and M1 are shown. The TGFα shedding assay was performed using HEK293 cells transfected with AP-TGFα, $G\alpha_{q/i1}$, and each GPR34-expressing vector. For negative control cells, empty plasmid was transfected instead of a receptor-expressing plasmid. In each panel, receptor-specific AP-TGFα release levels (differences in AP-TGFα release between receptor-expressing cells and negative control cells) are shown. Symbols and error bar mean average and SEM, respectively, from 5-8 independent experiments. See Supplementary Table 2 for parameters obtained from the concentration-responses curves. Source data are provided as a Source Data file.

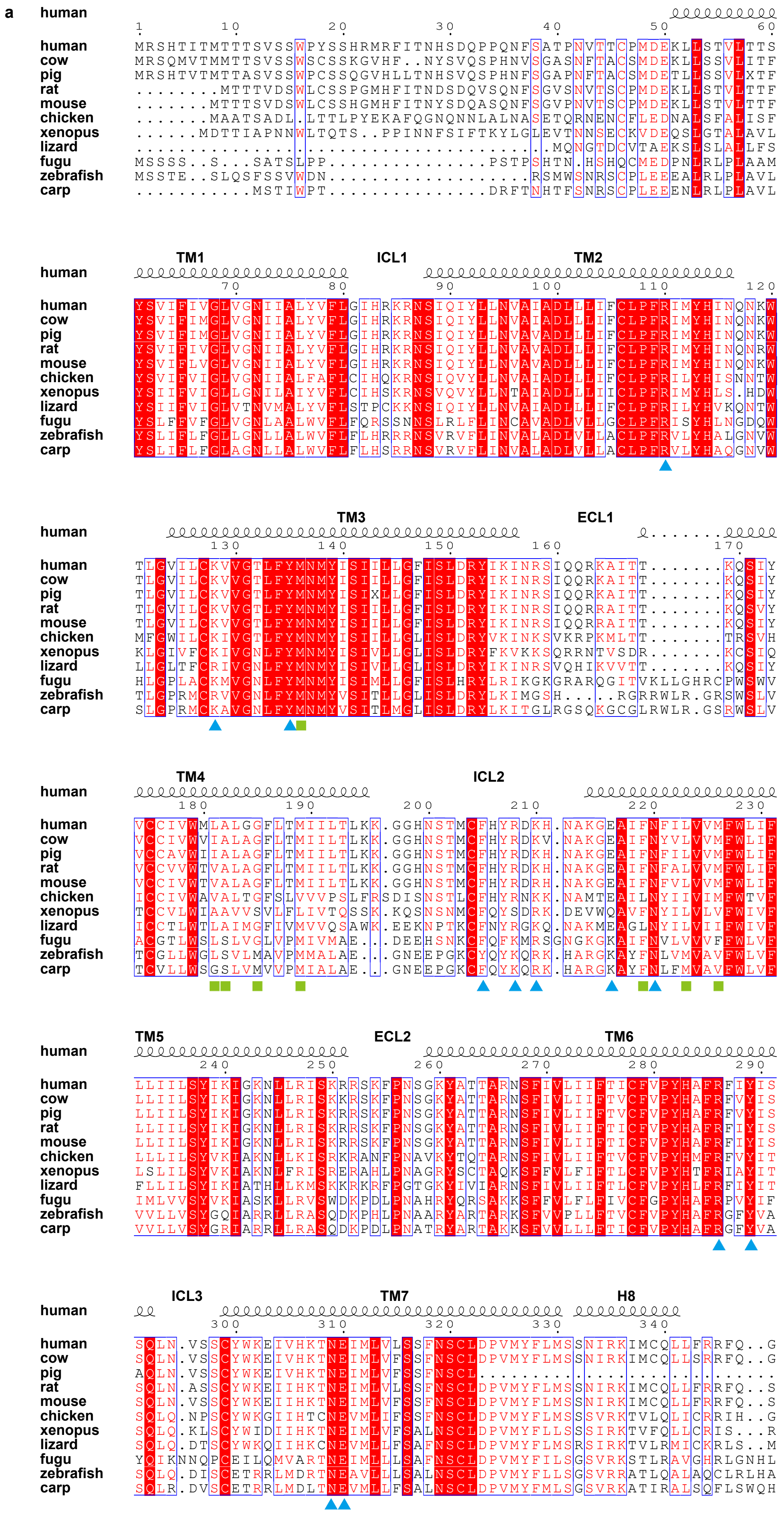

a
human
cow
pig
rat
mouse
chicken
xenopus
lizard
fugu
zebrafish
carp
TM1
ICL1
TM2
TM3
ECL1
TM4
ICL2
TM5
ECL2
TM6
ICL3
TM7
H8

```
human
           350       360       370       380
human      EPSRS.ESTSEFKPGYSLHDTSVAVKIQSSSKS.T.........................
cow        EASRS.ESTSEFKPGYSLHDTSAVAKIQTTS.............................
pig        ............................................................
rat        DTSRS.ESTSEFKPGYSLHDLSVTVKM.QYSTK.GN........................
mouse      EASRS.ESTSEFKPGHSLHDLSVTVKMPQYSTK.GN........................
chicken    DSSVTLESTSEIKLGQYMQERLSTTTPHSSSVKEKSDL..IK..................
xenopus    DTGRSESNTSELHQVHNLHESVCGPSHNNYSQSTTFFSRQLRTTVRNIR...........
lizard     ESNIS.DSTSEMKQRYSSRDHLESPNHHSRIYFIKNVK......................
fugu       LFLQDASSTTESRRPSGPVVLPNTPSVTHRTSMINNA..TLRRN................
zebrafish  EPPATNSSTTELRRTSVSHASTTAVLDTSRCSLGNIT..TLRSQHTDLHP..........
carp       EPAVTNSSTTELRRTSVSHTSTAAVLPTSRGSLGLIT..ASPCSKPDDRESYLEMTRIKL

human

human      .................
cow        .................
pig        .................
rat        .................
mouse      .................
chicken    .................
xenopus    .................
lizard     .................
fugu       .................
zebrafish  ....HSEPCVHRSGLSD
carp       HTFPNYAGTLPHSGL..
```

▲ : hydrophilic pocket

■ : hydrophobic pocket

b

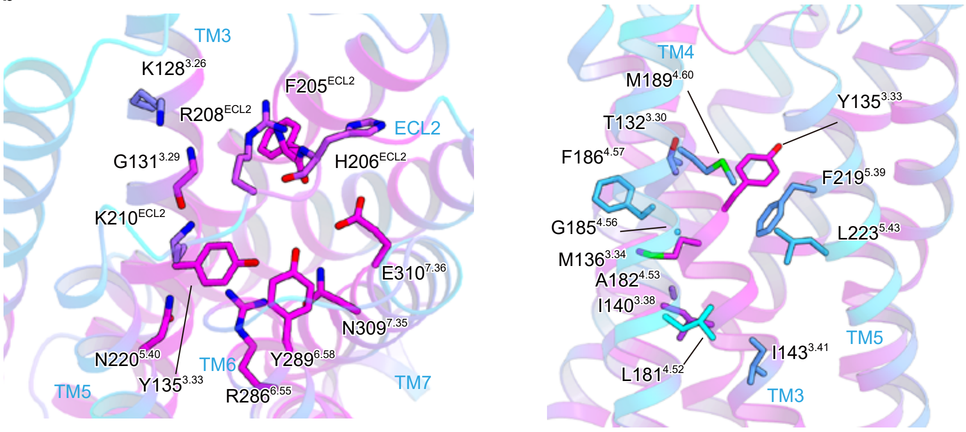

Supplementary Fig. 5 Alignment of GPR34 across vertebrates.

(a) Alignment of GPR34 across vertebrates. Amino acid sequence alignment of GPR34 from human (Uniprot ID: P0DMS8), cow (Uniprot ID: Q0VC81), pig (Uniprot ID: F1SBN8), rat (Uniprot ID: P28647), mouse (Uniprot ID: Q3U4C5), chicken (Uniprot ID: R4GIJ8), Xenopus (Uniprot ID: ENSXETT00000046736.1), lizard (Uniprot ID: G1KF19), and fugu (Uniprot ID: H2VBI6). Secondary structure elements for α-helices are indicated by cylinders. Conservation of the residues is indicated as follows: red panels for completely conserved; red letters for partially conserved; and black letters for not conserved.
(b) Conservation of the residues of GPR34. The sequence conservation among the vertebrate homologues of GPR34 was calculated using the ConSurf server (http://consurf.tau.ac.il) and is colored from cyan (low) to maroon (high).

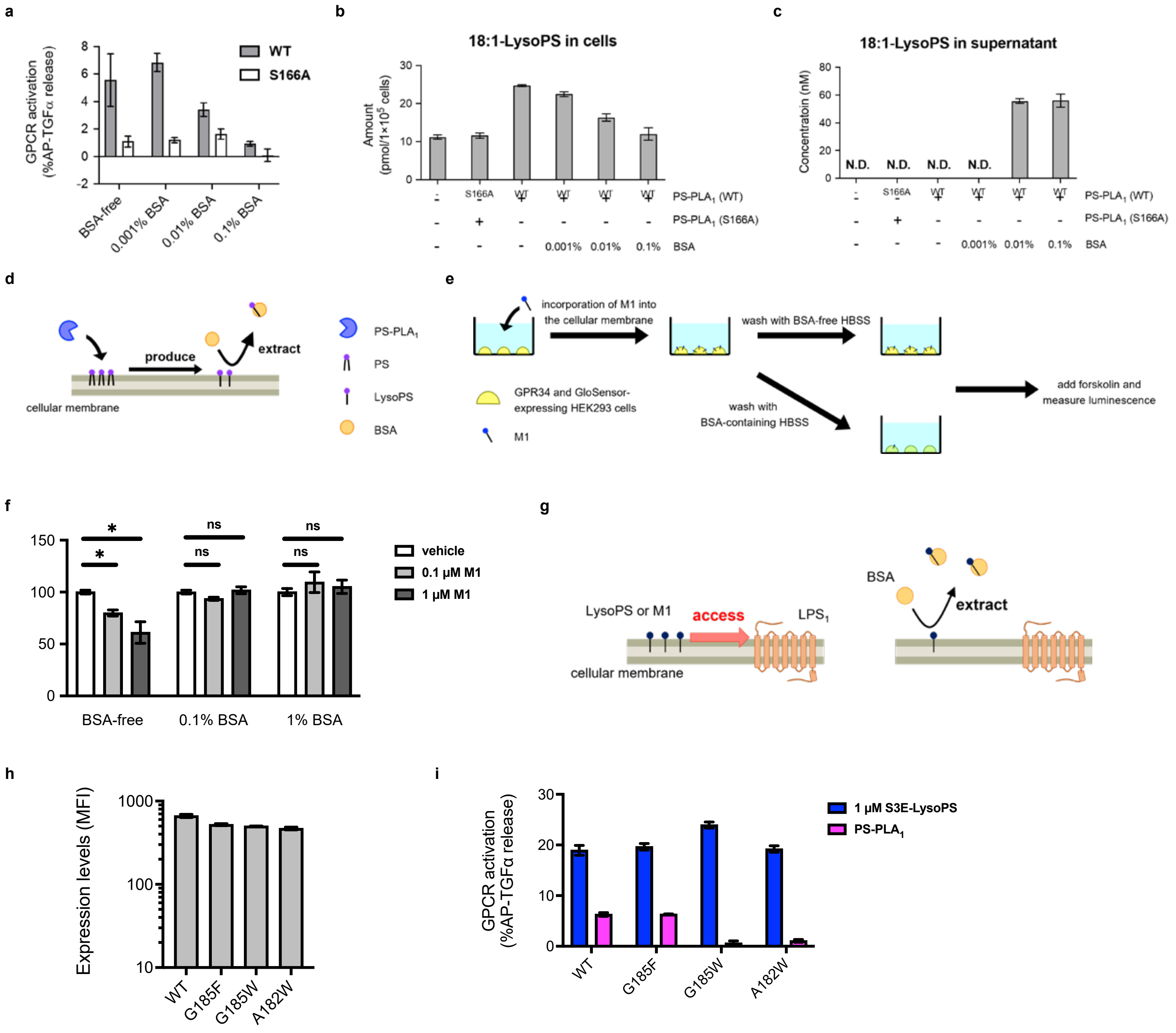

Supplementary Fig. 6 LysoPS and M1 access GPR34 from its lateral side.

(a) GPR34 activation induced by recombinant PS-PLA$_1$ and the effect of LysoPS extraction from the cellular membrane on it. HEK293A cells expressing AP-TGFα, G$\alpha_{q/i1}$, and GPR34 were stimulated by recombinant PS-PLA$_1$ with or without BSA. For the negative control, HEK293 cells transfected with empty plasmid instead of GPR34-encoding plasmid were used. After 1 hour incubation, the amount of AP-TGFα in the supernatant was determined. The receptor-specific AP-TGFα release (differences in AP-TGFα release between GPR34-expressing cells and negative control cells) are shown. S166A is a mutant PS-PLA$_1$, which do not have the catalytic activity. **(b and c)** The amount of 18:1-LysoPS in the cells treated with recombinant PS-PLA$_1$ and the concentration of 18:1-LysoPS in the supernatant. HEK293A cells were treated with recombinant PS-PLA$_1$ for 1 hour. Then, LysoPS in the cells and the supernatant was extracted into MeOH, and the amount was evaluated by LC-MS/MS. **(d)** The illustration of GPR34 activation induced by PS-PLA$_1$. PS-PLA$_1$ hydrolyzes phosphatidylserine in the cellular membrane to produce LysoPS. The LysoPS laterally spreads in the cellular membrane and accesses GPR34. **(e and f)** The effect of M1 incorporation into and extraction from the cellular membrane on GPR34 activation are shown. The schematic illustration of experimental procedure is shown **(e)**. HEK293 cells expressing GPR34 and GloSensor-22F were incubated with M1 and washed by BSA-free or BSA-containing HBSS. After that, forskolin was added and the luminescence was measured. The cAMP levels in the HEK293 cells-expressing are shown **(f)**. **(g)** The schematic illustration of M1 accessing GPR34. GPR34 can be activated by M1, which is in the cellular membrane. This is indicated by the data (e), which showed GPR34 was not activated when the cells were washed by BSA-containing HBSS and M1 was extracted from the cellular membrane. **(h)** The expression levels of the additional mutants (G185F, G185W, and A182W). **(i)** GPR34 activation of the additional mutants induced by S3E-LysoPS or PS-PLA$_1$.HEK293A cells expressing AP-TGFα, G$\alpha_{q/i1}$, and GPR34 were stimulated by S3E-LysoPS or PS-PLA$_1$. Source data are provided as a Source Data file.

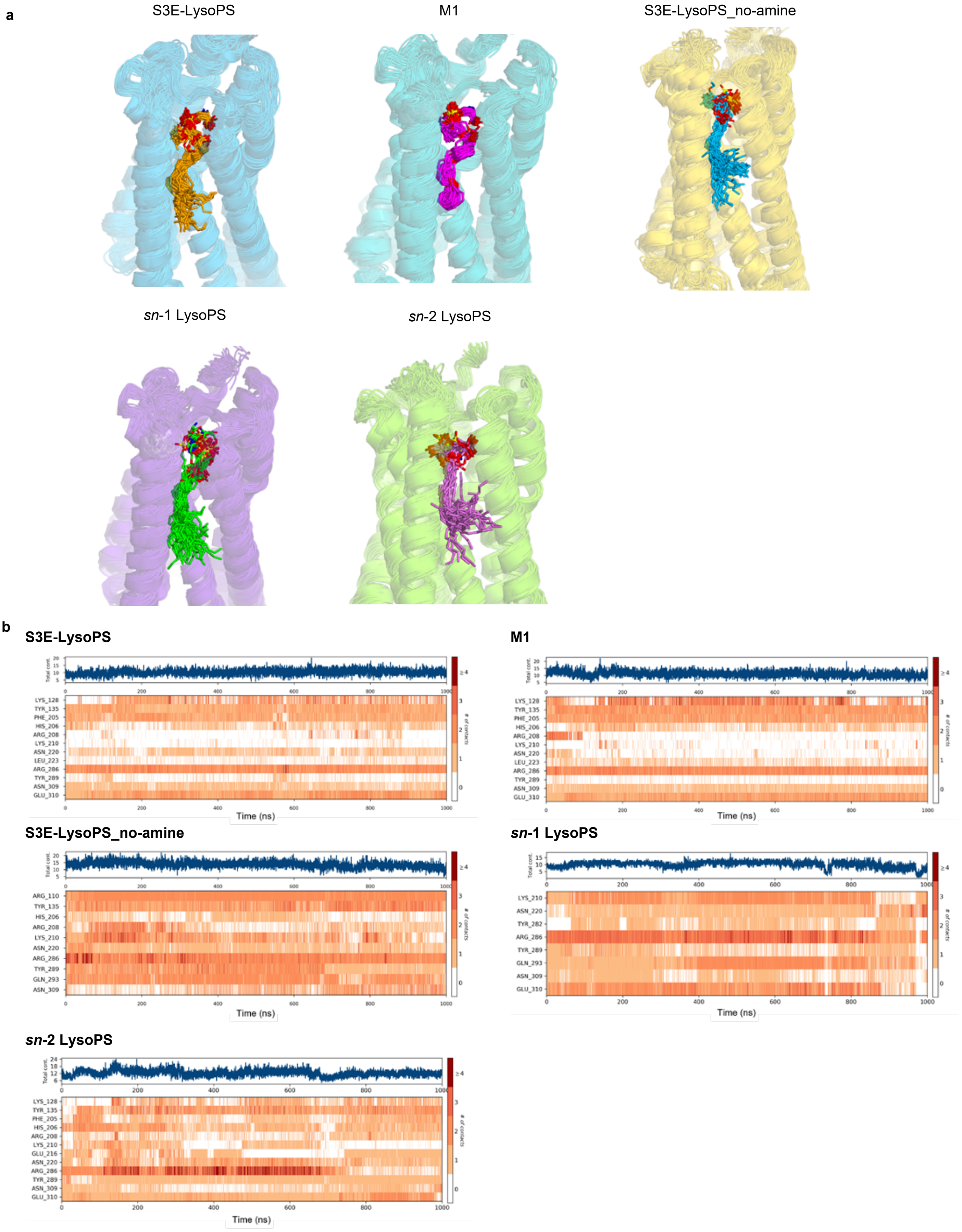
a
S3E-LysoPS
M1
S3E-LysoPS_no-amine
sn-1 LysoPS
sn-2 LysoPS
b
S3E-LysoPS
M1
S3E-LysoPS_no-amine
sn-1 LysoPS
sn-2 LysoPS
Total cont.
# of contacts
LYS_128
TYR_135
PHE_205
HIS_206
ARG_208
LYS_210
ASN_220
LEU_223
ARG_286
TYR_289
ASN_309
GLU_310
ARG_110
GLN_293
TYR_282
GLU_216
Time (ns)

c

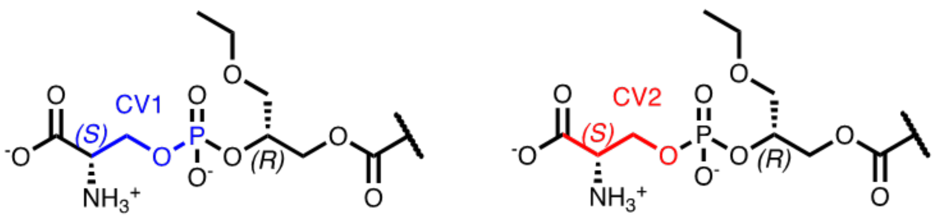

d M1

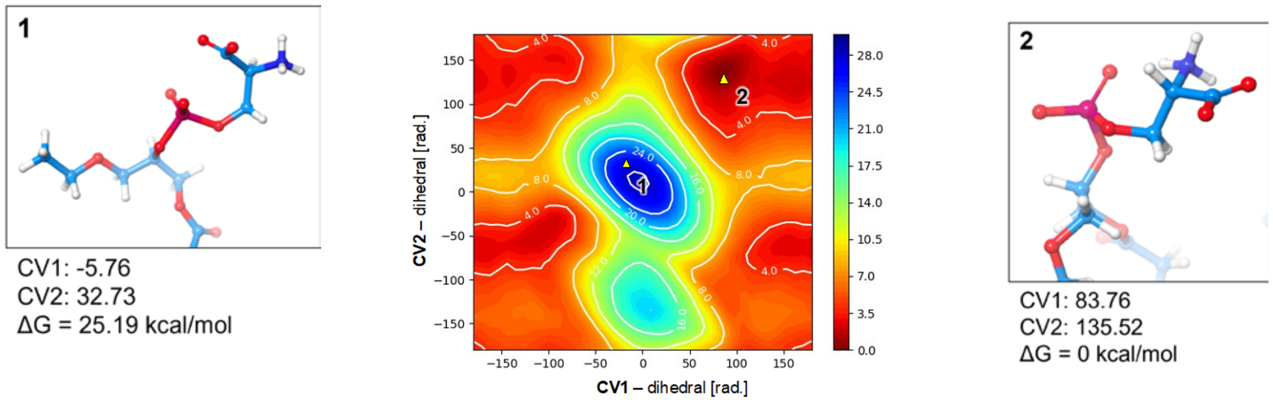

e S3E-LysoPS

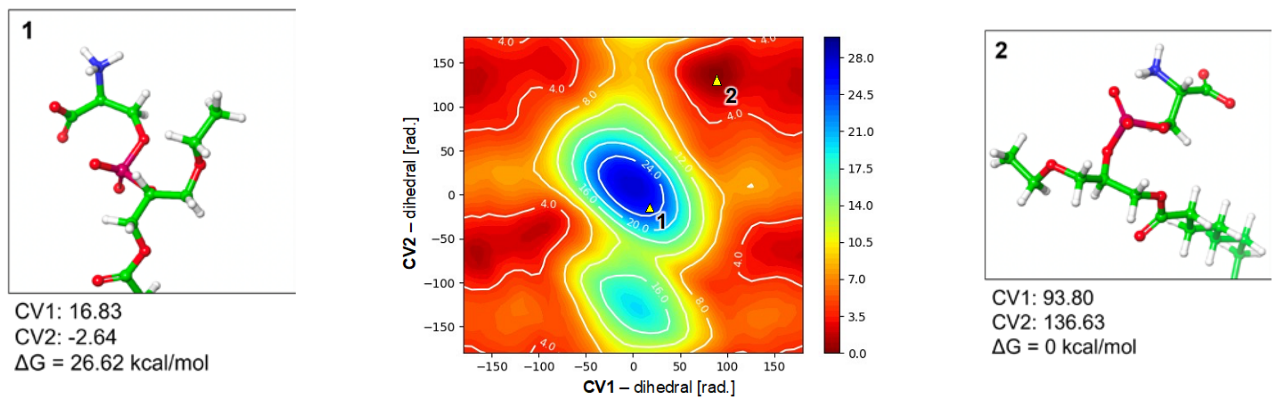

f M1

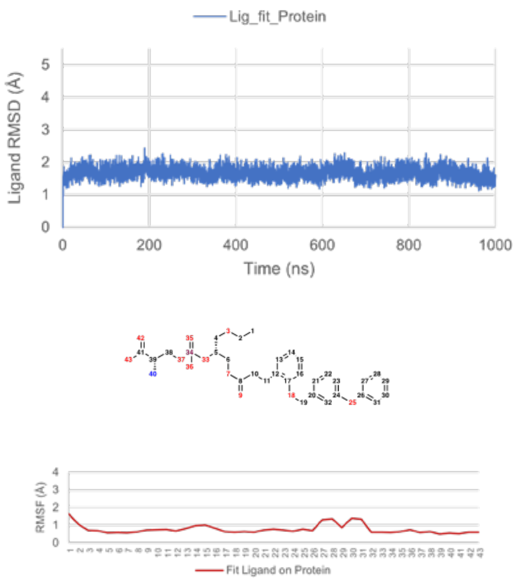

g S3E-LysoPS

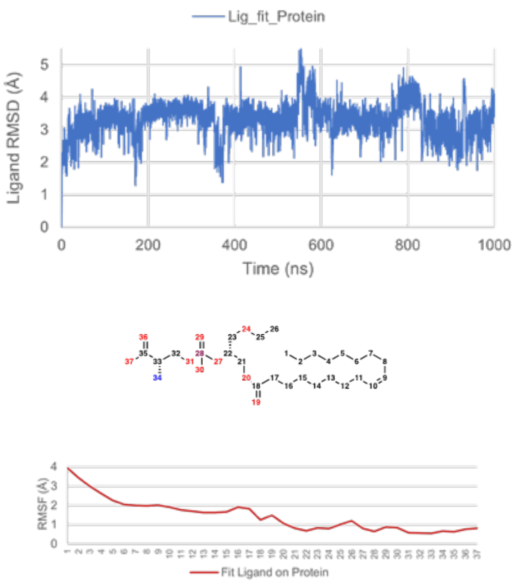

h M1

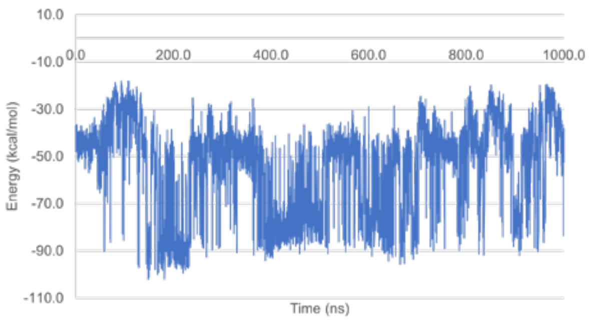

i S3E-LysoPS

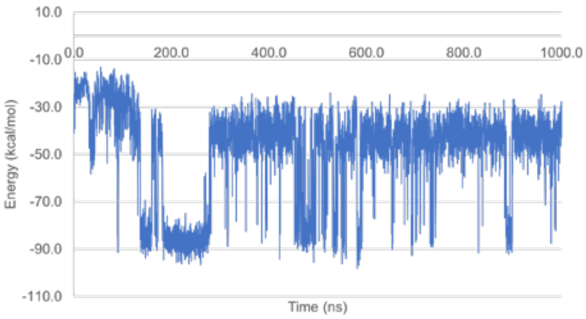

j **M1**

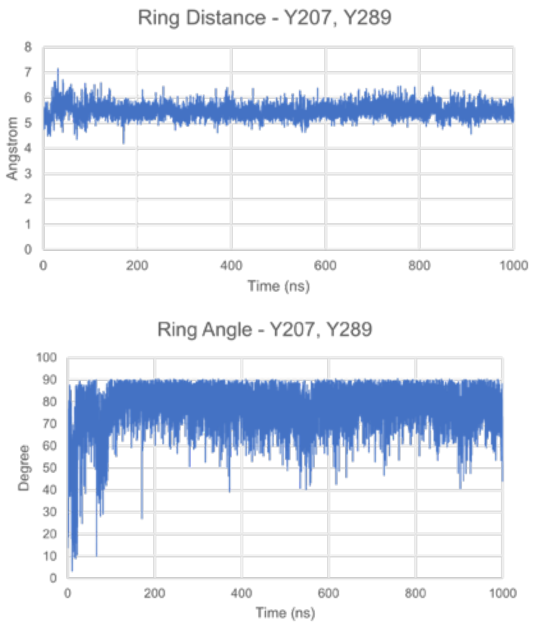

k **S3E-LysoPS**

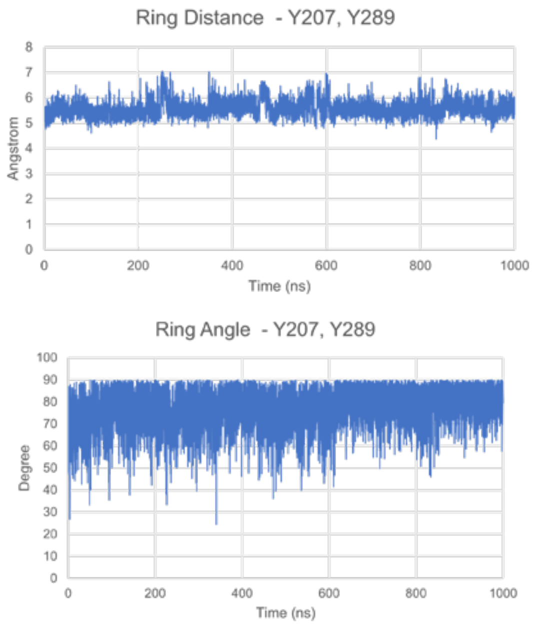

l

sn-1 derivative
(deoxy-*sn*-1-C3-Ph-*o*-OBn-*m*-OTol)

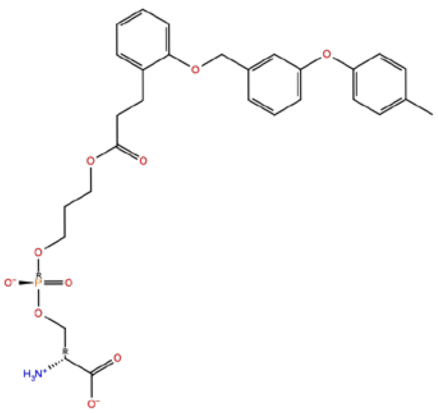

M1-GPR34 (MD) *sn*-1 derivative-GPR34 (docking)

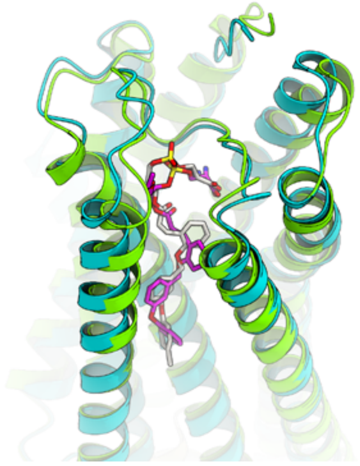

Supplementary Fig. 7 Molecular dynamics simulations.

(a) Ensembles from each trajectory. The complex structures were extracted every 20 ns and superimposed on the first structure. **(b)** A timeline representation of the interactions and contacts. The top panel shows the total number of contacts over the whole trajectory. The bottom panel shows residue-specified interact of the ligand in each trajectory frame (darker color represents larger contact count). **(c)** Definition of CV1 and CV2 in metadynamics simulation. **(d and e)** Energy landscape of **(d)** M1 and **(e)** S3E-LysoPS in metadynamics simulation, and representative conformations, including energetically unstable conformation **1**, and energetically stable conformation **2** (U-shape). **(f and g)** Ligand RMSD (top panel) and RMSF values (bottom panel) during the 1 μs MD simulation. Atom index is illustrated on the chemical structure. **(h and i)** Ligand-K128 interaction energy calculated from 1 μs MD of **(h)** M1 and **(i)** S3E-LysoPS bound GPR34 (FF: OPLS3e). The interaction between M1 and K128 showed stronger binding (lower interaction energy) in a higher frequency as compared with S3E-LysoPS. **(j and k)** Ring distance and angle between Y207and Y289 in MD simulation of **(j)** M1 and **(k)** S3E-LysoPS bound GPR34. **(l)** Docking pose of an *sn*-1 deoxyLysPS derivative, deoxy-*sn*-1-C3-Ph-*o*-OBn-*m*-OTol.

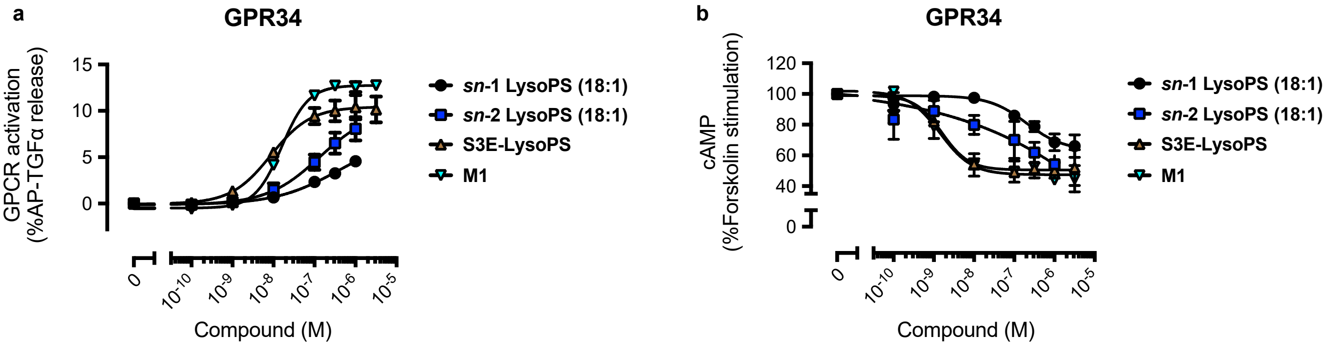

c **TGFα shedding assay.**

Compound	pEC_{50}	EC_{50}	E_{max}	*n* =
sn-1 LysoPS (18:1)	6.45 ± 0.25	350 nM	7.5 ± 1.2%	3
sn-2 LysoPS (18:1)	6.52 ± 0.09	300 nM	13 ± 1.5%	3
S3E-LysoPS	8.03 ± 0.02	9.4 nM	11 ± 0.3%	3
M1	7.77 ± 0.01	17 nM	14 ± 0.1%	3

d **cAMP assay.**

Compound	pEC_{50}	EC_{50}	E_{max}	*n* =
sn-1 LysoPS (18:1)	< 5.5	> 3 μM	NA	3
sn-2 LysoPS (18:1)	6.85 ± 0.06	140 nM	10 ± 1.3%	3
S3E-LysoPS	8.07 ± 0.04	8.5 nM	10 ± 0.7%	3
M1	7.80 ± 0.03	16 nM	13 ± 0.4%	3

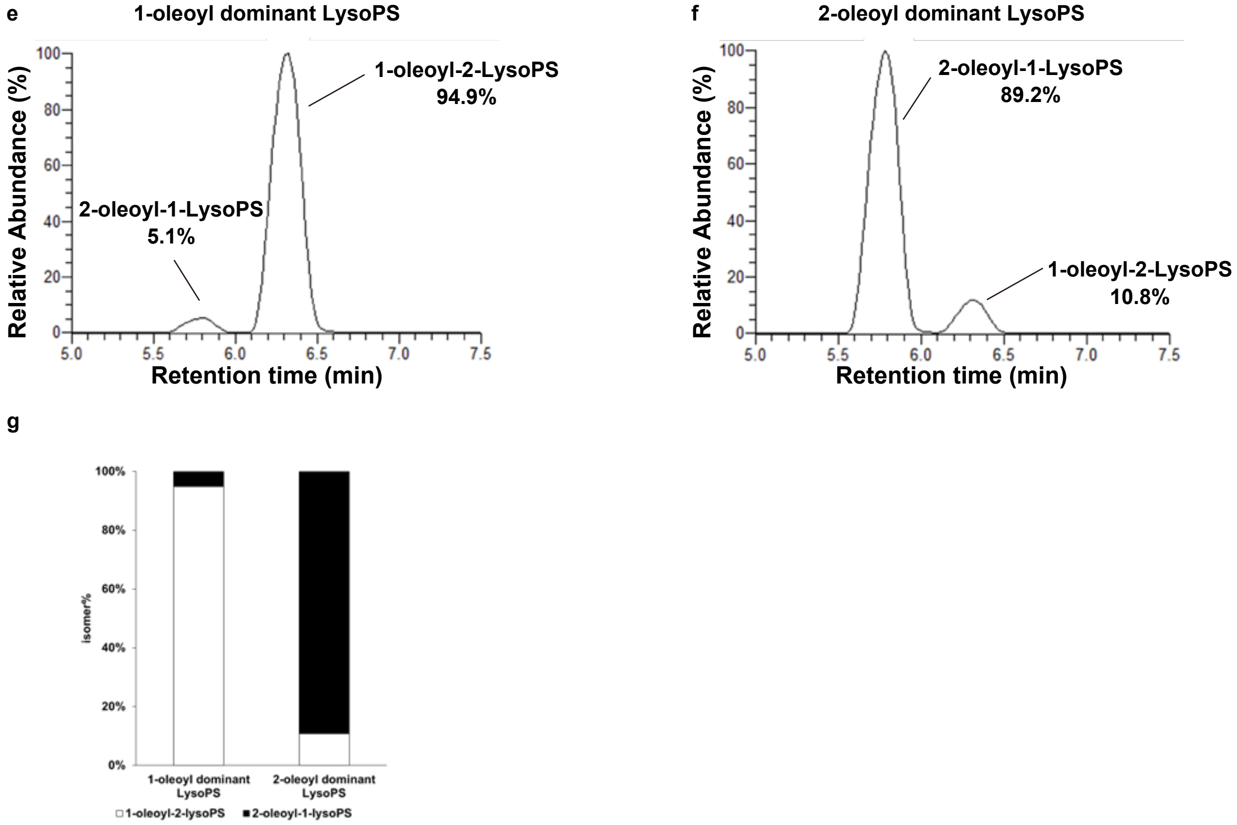

Supplementary Fig. 8 Purity of *sn*-1 and *sn*-2 LysoPS preparation used in this study.

(a and b) Agonist activities of each lysophosphatidylserine (LysoPS) molecule toward GPR34, measured by the transforming growth factor (TGF)-α shedding assay **(a)** and the cyclic adenosine monophosphate (cAMP) assay **(b)**. Both *sn*-1 LysoPS (18:1) and *sn*-2 LysoPS (18:1) were prepared by digesting di-oleoyl (18:1) PS with Rhizomucor miehei lipase, which has phospholipase A_1 activity. For the TGF-α shedding assay (b), HEK293A cells expressing alkaline phosphatase-tagged (AP)-TGFα, $G\alpha_{q/i1}$, and GPR34, as well as negative-control cells transfected with empty plasmid in place of the GPR34-encoding plasmid, were treated with each LysoPS. After 1 h incubation, the amount of AP-TGFα in the supernatant was determined. Receptor-specific AP-TGFα release, measured as the difference in AP-TGFα release between GPR34-expressing and negative control cells, is shown for each agonist. In the cAMP assay (b), HEK293 cells expressing GPR34 and GloSensor-22F, a cAMP biosensor, were loaded with D-Luciferin and treated with each LysoPS plus forskolin. Luminescence signals were then measured and normalized, and the ratios of cAMP signal when stimulated with LysoPS vs. forskolin only are shown. Symbols and error bars indicate the average and standard error of the mean (SEM), respectively, from three independent experiments. **(c and d)** Parameters obtained from the TGF-α shedding assay **(c)** and the cAMP assay **(d)**. **(e and f)** Purity of sn-1 and sn-2 LysoPS preparation used in this study **(e)** Purity of 1-oleoyl-rich (A) and 2-oleoyl-rich LysoPS (B) was checked by LC-MS/MS analysis in which they were separated on LC. The chromatograms show a purity of about 95% for 1-oleoyl-rich LysoPS and about 90% for 2-oleoyl-rich LysoPS. **(f)** Quantification data for (e and f) are shown in **(g)**. Source data are provided as a Source Data file.

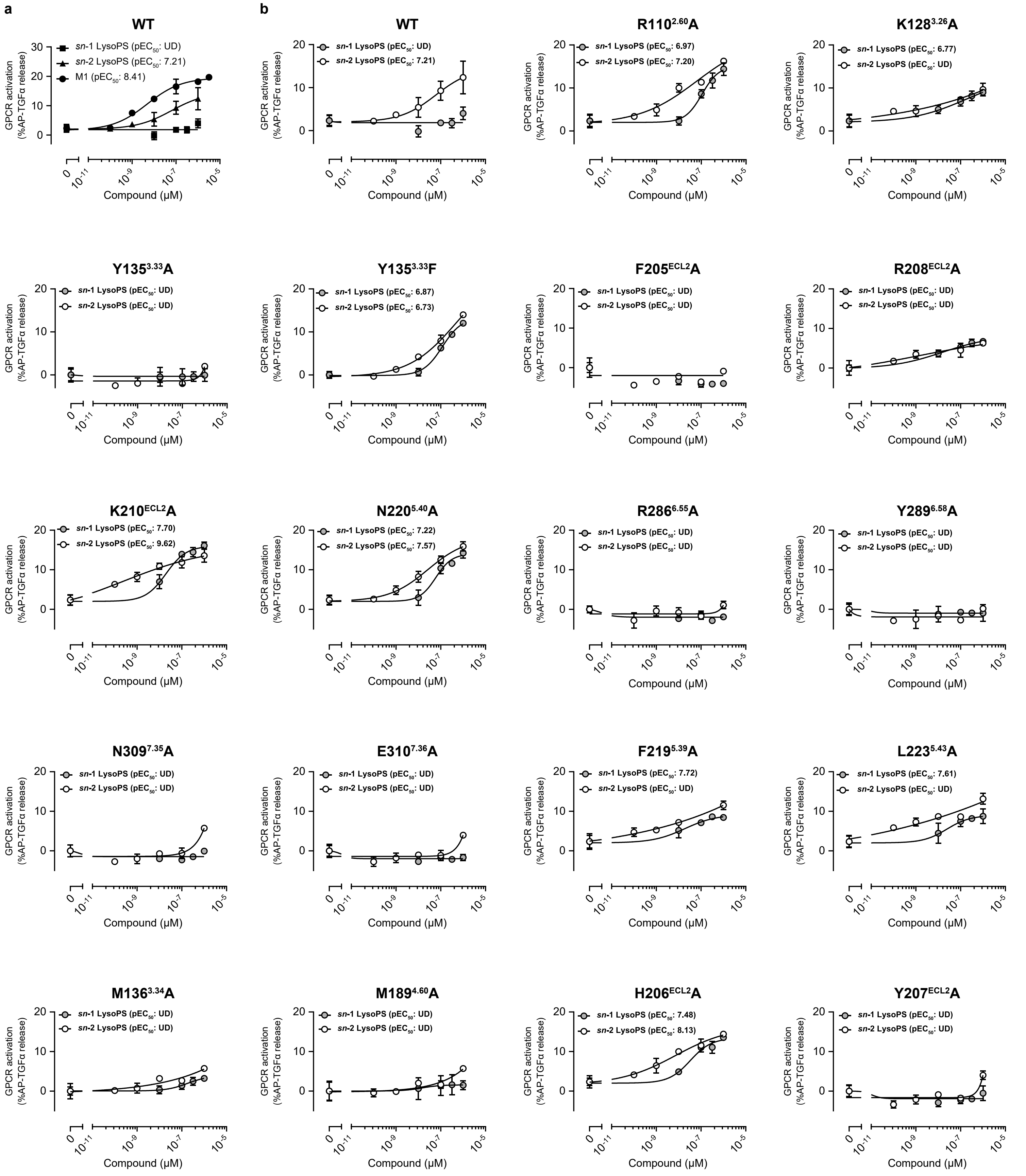

Supplementary Fig. 9 The responses of GPR34 toward *sn*-1 LysoPS and *sn*-2 LysoPS.

(a) The response of wild-type GPR34 toward *sn*-1 LysoPS, *sn*-2 LysoPS, and M1 is shown. The TGFα shedding assay was performed using HEK293 cells transfected with AP-TGFα, $G\alpha_{q/i1}$, and each GPR34-expressing vector. For negative control cells, empty plasmid was transfected instead of a receptor-expressing plasmid. In each panel, receptor-specific AP-TGFα release levels (differences in AP-TGFα release between receptor-expressing cells and negative control cells) are shown. Symbols and error bar mean average and SEM, respectively, from 5-8 independent experiments. **(b)** The responses of wild-type GPR34 and 18 mutants of GPR34 toward *sn*-1 LysoPS and *sn*-2 LysoPS are shown. The *sn*-1 response to the wild-type receptor was too low to calculate an EC_{50}, so we could only qualitatively distinguish a decrease in the *sn*-1 response due to mutation. Interestingly, some mutants had an increased *sn*-1 response, comparable to the *sn*-2 response. The specific reason for the result of mutagenesis is unclear, but it is likely that the ligand-binding pocket of GPR34 is optimized for recognition of *sn*-2 LysoPS. Source data are provided as a Source Data file.

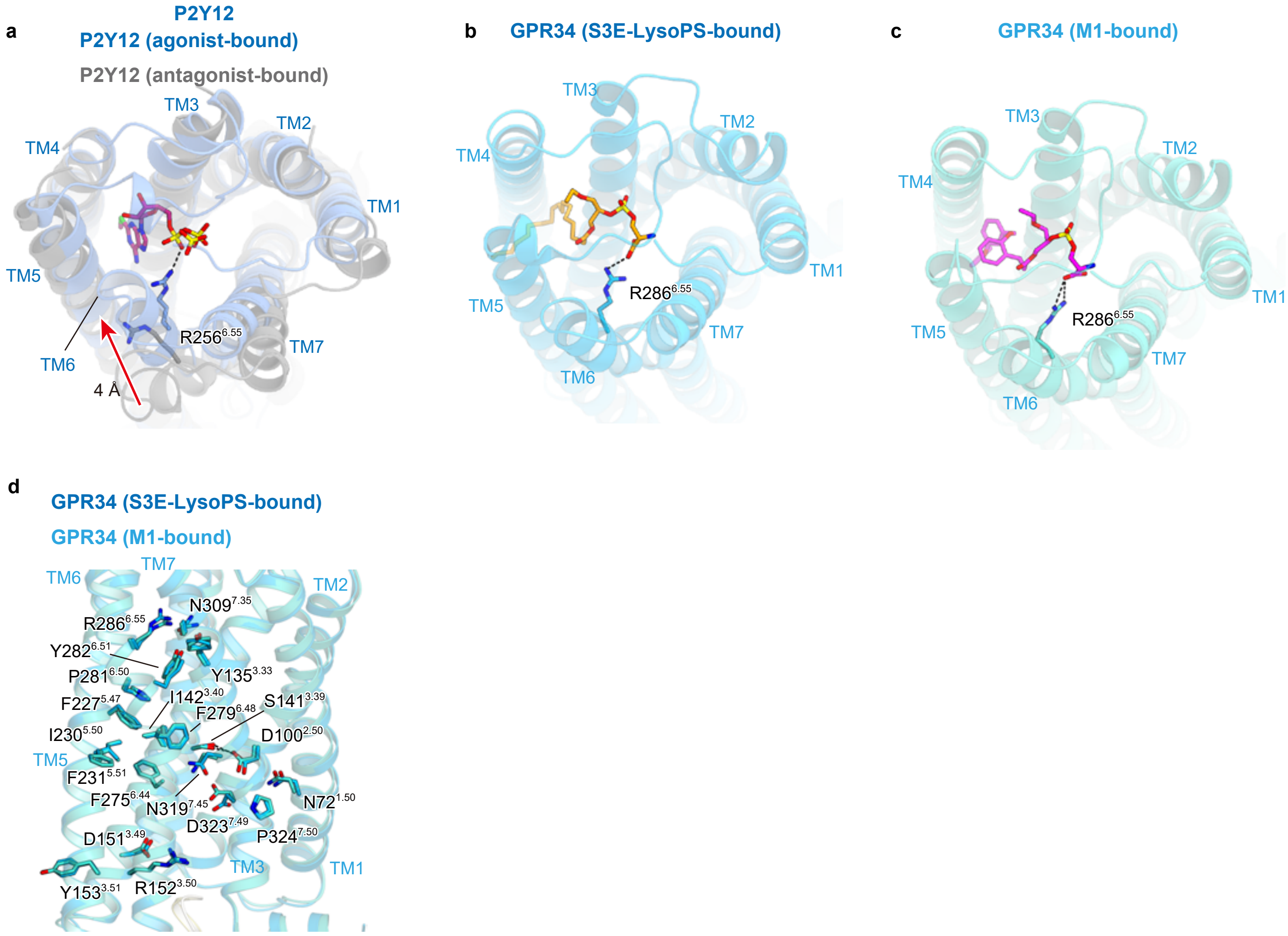

Supplementary Fig. 10 Comparison of the conserved motifs.

(a-c) Comparison of R$^{6.55}$ in P2Y12 **(a)** (PDB 4NTJ and 4PY0), S3E-LysoPS-bound-GPR34 **(b)**, and M1-bound GPR34 **(c)** structures. **(d)** Comparison of the conserved motifs at the receptor cores in the M1- and S3E-LysoPS-bound receptors.

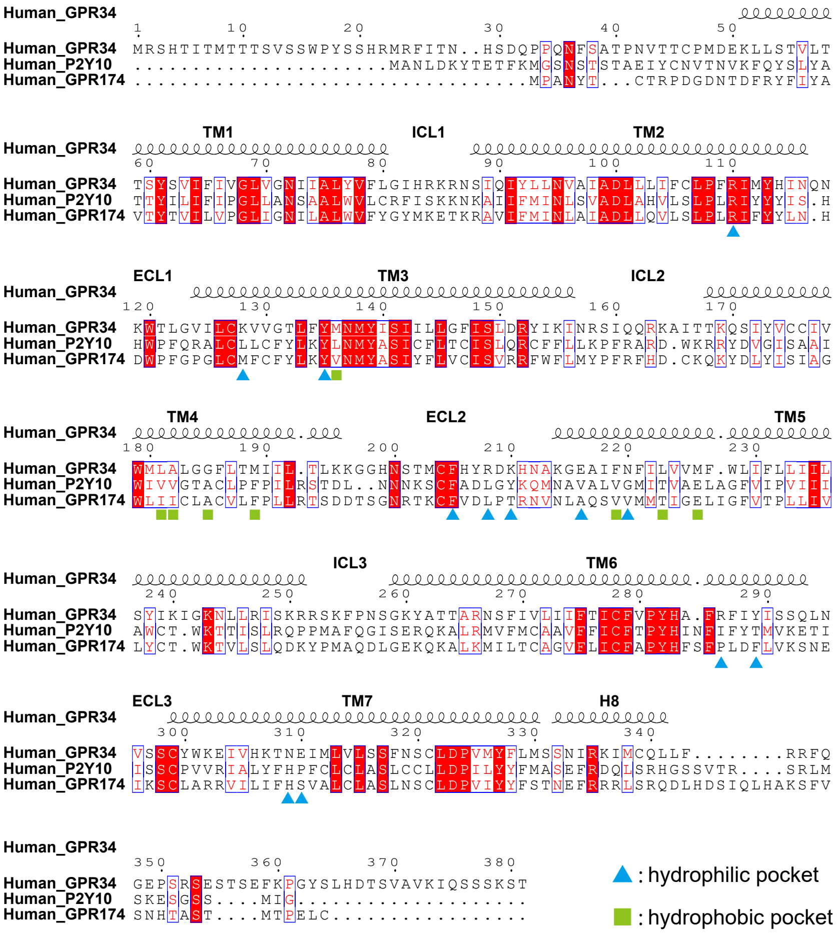

Supplementary Fig. 11 Comparison of the GPR34, P2Y10, and GPR174 sequences.

Alignment of the amino acid sequences of human GPR34 (Uniprot ID: Q9UPC5), human P2Y10 (Uniprot ID: O00398), and human GPR174 (Uniprot ID: Q9BXC1). Secondary structure elements for α-helices are indicated by cylinders. Conservation of the residues is indicated as follows: red panels for completely conserved, red letters for partially conserved, and black letters for not conserved.

Supplementary Discussion

Insight into ethoxy substitution

Intriguingly, although our structure–function relationship (SAR) results suggest enhanced activity by introducing an ethoxy group at the *sn*-1 position, the populated positions of the ethoxy substituent in M1 (alongside TM4) and S3E-LysoPS (pointing at TM5) are similar (Fig. 4a,b, Supplementary Fig. 7a, and Supplementary Video 1). In both the cryo-EM structures and MD simulations, the ethoxy group does not make apparent interactions with receptor residues (Fig. 2e, 3b, and 4a,b). Instead, this pinning ethoxy group tightly restricts and stabilizes the overall binding poses of the ligands (M1 and S3E-LysoPS), including both the hydrophilic moiety (root-mean-square-fluctuation [RMSF]P <1 Å, [RMSF]Ser <1 Å) and hydrophobic moiety (Supplementary Fig. 7f,g), by filling up the space in the vicinity of TMs 4–6 and extracellular loop (ECL)2.

Insight into the MD simulation of the S3E-LysoPS- and M1-bound receptors

The results of the mutant experiments are in rough agreement with the residues observed to interact in the MD simulation, but attention should be paid to K128$^{3.26}$ and Y289$^{6.58}$. The K128A mutation did not affect the potency of S3E-LysoPS, but greatly reduced that of M1 (Supplementary Table 2), and even though the interaction between K128A and the ligand is conserved in both MD simulations of the S3E-LysoPS- and M1-bound GPR34 structures (Fig. 4a,b), the interaction fraction of S3E-LysoPS is lower than that of M1 (approximately 1.2 (S3E-LysoPS) vs. 1.6 (M1)). Besides, the calculated interaction energy (Force field: OPLS3e) throughout the trajectory suggested that the binding percentage between M1 and K128$^{3.26}$ is larger than that between S3E-LysoPS and K128$^{3.26}$ (Supplementary Fig. 7h,i), consistent with the mutational analysis. As for Y289$^{6.58}$, we found that it formed hydrophilic interactions with the serine moieties of ligands in both the M1- and S3E-LysoPS-bound GPR34 structures (Fig. 4a,b). These interactions are stable, but occur in a low-frequency manner. In addition to directly contacting the ligand, Y289$^{6.58}$ also stabilizes the binding pocket by tilted T-shaped pi-pi stacking with Y207^{ECL2} (Supplementary Fig. 7j,k). Thus, the Y289A mutant should have dramatically reduced activity in the mutagenesis study (Supplementary Table 2).

Docking of *sn*-1 LysoPS derivative

In our previous studies of LysoPS derivatives, we could increase ligand activity of an sn-1-type derivative to a level comparable to that of *sn*-3 ligands by properly modifying the structure of the fatty acid moiety (Supplementary Fig. 7k). In this sense, the ligand' s hydrophobic tail can play a role similar to that of the ethoxy group in S3E-LysoPS, enabling the hydrophilic regions of *sn*-1-type ligands to adopt the proper U-shaped conformation required to interact with the binding site in a manner similar to *sn*-3-type derivatives (Supplementary Fig. 7l). This implies that structural change in the glycerol linker (e.g., S3E-LysoPS), as well as in the hydrophobic region, can induce the proper conformational changes in the phosphoserine part of the molecule, facilitating interaction with the binding pocket of GPR34, and thus activating GPR34 activity[1] .

Insight into the D151A mutational effect

The D151A mutation of GPR34 has been previously associated with lymphoma due to its capacity to augment G-protein activity[2]. In the present cryo-EM structures, D151$^{3.49}$ does not directly engage with G_i (Fig. 5c). D151$^{3.49}$ constitutes an essential part of the conserved DRY motif, wherein D$^{3.49}$ establishes an ionic lock with R$^{3.50}$, impeding its structural transition during receptor activation. Observing that R152$^{3.50}$ is similarly oriented toward the center of the transmembrane bundle, as observed in other GPCR-G protein complexes (Fig. 5c), the ionic lock between D151$^{3.49}$ and R152$^{3.50}$ likely exists in the inactive state of GPR34. Consequently, the disruption of this ionic lock could elucidate the heightened activity resulting from the D151A mutation.

Supplementary Reference

1. Jung, S. et al. Identification of Bioactive Conformational Space of Flexible Endogenous Lipid Mediator Lysophosphoserine Based on Accessible Spaces and Activities of Conformationally Restricted Analogues. (2022) doi:10.26434/chemrxiv-2022-bfgzt.
2. Korona, B., Korona, D., Zhao, W., Wotherspoon, A. C. & Du, M.-Q. GPR34 activation potentially bridges lymphoepithelial lesions to genesis of salivary gland MALT lymphoma. Blood 139, 2186–2197 (2022).
