# Supplementary material for: Structural basis for lysophosphatidylserine recognition by GPR34
Source: Nat Commun. 2024 Feb 7;15:902. doi: 10.1038/s41467-024-45046-z (PMC10850092; doi:10.1038/s41467-024-45046-z)
Supplement: Supplementary file 3 — Description of Additional Supplementary Files [file 41467_2024_45046_MOESM3_ESM.pdf]

File name: Supplementary Movie 1

Description: 1-µs molecular dynamics (MD) simulation of GPR34 bound to the various agonists. The MD simulation movie was recorded every 500 ps. Hydrophilic network residues (*e.g.,* K128$^{3.26}$, F205^{ECL2}, R286$^{6.55}$, N309$^{7.35}$, and E310$^{7.36}$) are shown as sticks. The hydrophilic binding sites of GPR34 contain an assembly of charged amino acid residues that can efficiently interact with the charged phosphoserine. Therefore, hydrophilic interactions of the ligand in the binding pocket could be swapped. In fact, in the 1-µs MD simulation of *sn*-1 LysoPS, the *sn*-1-type hydrophilic interaction mode barely switched to the *sn*-3-type, consistent with its low activity. The movies of the five simulations are concatenated as one movie, S3E-LysoPS, M1, S3E-LysoPS-no_amine, *sn*-1 LysoP1, and *sn*-2 LysoPS, in that order.
