# Supplementary material for: Structural basis for lysophosphatidylserine recognition by GPR34
Source: Nat Commun. 2024 Feb 7;15:902. doi: 10.1038/s41467-024-45046-z (PMC10850092; doi:10.1038/s41467-024-45046-z)
Supplement: Supplementary file 5 — Reporting Summary [file 41467_2024_45046_MOESM5_ESM.pdf]

nature portfolio

Corresponding author(s): Osamu Nureki

Last updated by author(s): Dec 7, 2023

Reporting Summary

Nature Portfolio wishes to improve the reproducibility of the work that we publish. This form provides structure for consistency and transparency in reporting. For further information on Nature Portfolio policies, see our Editorial Policies and the Editorial Policy Checklist.

Statistics

For all statistical analyses, confirm that the following items are present in the figure legend, table legend, main text, or Methods section.

n/a	Confirmed	
☐	☒	The exact sample size (*n*) for each experimental group/condition, given as a discrete number and unit of measurement
☐	☒	A statement on whether measurements were taken from distinct samples or whether the same sample was measured repeatedly
☐	☒	The statistical test(s) used AND whether they are one- or two-sided<br>*Only common tests should be described solely by name; describe more complex techniques in the Methods section.*
☒	☐	A description of all covariates tested
☒	☐	A description of any assumptions or corrections, such as tests of normality and adjustment for multiple comparisons
☐	☒	A full description of the statistical parameters including central tendency (e.g. means) or other basic estimates (e.g. regression coefficient) AND variation (e.g. standard deviation) or associated estimates of uncertainty (e.g. confidence intervals)
☐	☒	For null hypothesis testing, the test statistic (e.g. *F*, *t*, *r*) with confidence intervals, effect sizes, degrees of freedom and *P* value noted<br>*Give P values as exact values whenever suitable.*
☒	☐	For Bayesian analysis, information on the choice of priors and Markov chain Monte Carlo settings
☒	☐	For hierarchical and complex designs, identification of the appropriate level for tests and full reporting of outcomes
☒	☐	Estimates of effect sizes (e.g. Cohen's *d*, Pearson's *r*), indicating how they were calculated

Our web collection on statistics for biologists contains articles on many of the points above.

Software and code

Policy information about availability of computer code

Data collection	SerialEM, Maestro2019-3
Data analysis	RERION3.1, PHENIX-1.14-3260, COOT-0.9.6, UCSF Chimera 1.15, UCSF ChimeraX 1.2.5, Cuemol2 2.2.3.443, Prism 7, VMD1.9.4a57

For manuscripts utilizing custom algorithms or software that are central to the research but not yet described in published literature, software must be made available to editors and reviewers. We strongly encourage code deposition in a community repository (e.g. GitHub). See the Nature Portfolio guidelines for submitting code & software for further information.

Data

Policy information about availability of data

All manuscripts must include a data availability statement. This statement should provide the following information, where applicable:

- Accession codes, unique identifiers, or web links for publicly available datasets
- A description of any restrictions on data availability
- For clinical datasets or third party data, please ensure that the statement adheres to our policy

Density maps and structure coordinates have been deposited in the Electron Microscopy Data Bank (EMDB) and the PDB, with accession codes EMD-38215 and PDB 8XBE for the S3E-LysoPS-GPR34-Gi complex; EMD-38217 and PDB 8XBG for the S3E-LysoPS-GPR34-Gi complex (Receptor focused); EMD-38218 and PDB 8XBH for the M1-GPR34-Gi complex; EMD-38219 and PDB 8XBI for the M1-GPR34-Gi complex (Receptor focused). The dynamics data and the simulation protocols were

uploaded in GPCRmd (www.gpcrmd.org), with dynamic ids 1741 for the S3E-LysoPS-GPR34 complex; 1742 for the M1-GPR34 complex; 1743 for the sn1-18:1 LysoPS-GPR34 complex; 1744 for the sn2-18:1 LysoPS-GPR34 complex, and 1745 for the S3E-LysoPS_noamine-GPR34 complex.

Research involving human participants, their data, or biological material

Policy information about studies with human participants or human data. See also policy information about sex, gender (identity/presentation), and sexual orientation and race, ethnicity and racism.

Reporting on sex and gender	N/A
Reporting on race, ethnicity, or other socially relevant groupings	N/A
Population characteristics	N/A
Recruitment	N/A
Ethics oversight	N/A

Note that full information on the approval of the study protocol must also be provided in the manuscript.

Field-specific reporting

Please select the one below that is the best fit for your research. If you are not sure, read the appropriate sections before making your selection.

☒ Life sciences ☐ Behavioural & social sciences ☐ Ecological, evolutionary & environmental sciences

For a reference copy of the document with all sections, see nature.com/documents/nr-reporting-summary-flat.pdf

Life sciences study design

All studies must disclose on these points even when the disclosure is negative.

Sample size	Sample sizes were not predetermined by statistical methods. For cryo-EM data, sample size were determined by availability of microscope and images were collected until the resolution and 3D reconstruction converges. For all the functional assay, we use sample size at least of three independent experiments, commonly exploited by researches in this field.
Data exclusions	No data were excluded from the analysis.
Replication	Protein samples were purified from different purification batch and used for the cryo-EM study. The biochemical experiments in this study have been repeated by greater than or equal to three independent experiments, and those findings are reliably reproduced.
Randomization	Randomization was not relevant to this study, as the data were collected automatically and did not involve choosing.
Blinding	Blinding is not necessary or valid for the purposes of structural determination. For cryo-EM study, imaging data were collected automatically. For functional analysis, blinding was not necessary by reason of the quantitative nature of the experiment.

Reporting for specific materials, systems and methods

We require information from authors about some types of materials, experimental systems and methods used in many studies. Here, indicate whether each material, system or method listed is relevant to your study. If you are not sure if a list item applies to your research, read the appropriate section before selecting a response.

Materials & experimental systems

n/a	Involved in the study	
☐	☒	Antibodies
☐	☒	Eukaryotic cell lines
☒	☐	Palaeontology and archaeology
☐	☒	Animals and other organisms
☒	☐	Clinical data
☒	☐	Dual use research of concern
☒	☐	Plants

Methods

n/a	Involved in the study	
☒	☐	ChIP-seq
☐	☒	Flow cytometry
☒	☐	MRI-based neuroimaging

Antibodies

Antibodies used	Anti-Flag M1 resin (Merck), goat anti-mouse IgG conjugated with Alexa488 (Thermo Fisher Scientific).
Validation	The antibodies used are commercially purchased and have been validated by the vendors. Validation data are available from the vendor's website.

Eukaryotic cell lines

Policy information about cell lines and Sex and Gender in Research

Cell line source(s)	Sf9 insect cells (Thermo Fischer Scientific), HEK293A cells (Thermo Fischer Scientific)
Authentication	None of cell lines were authenticated.
Mycoplasma contamination	Not tested.
Commonly misidentified lines (See ICLAC register)	None of commonly misidentified lines were used in this study.

Animals and other research organisms

Policy information about studies involving animals; ARRIVE guidelines recommended for reporting animal research, and Sex and Gender in Research

Laboratory animals	This study used C57BL/6J mice. Number, age, and genotype of mice used for the experiment are stated in the Methods section.
Wild animals	Not used.
Reporting on sex	Male mice were used.
Field-collected samples	Not used.
Ethics oversight	The animal experiment was approved by the animal ethics committee of the University of Tokyo prior to their commencement and performed in accordance with approved protocols.

Note that full information on the approval of the study protocol must also be provided in the manuscript.

Plants

Seed stocks	*Report on the source of all seed stocks or other plant material used. If applicable, state the seed stock centre and catalogue number. If plant specimens were collected from the field, describe the collection location, date and sampling procedures.*
Novel plant genotypes	*Describe the methods by which all novel plant genotypes were produced. This includes those generated by transgenic approaches, gene editing, chemical/radiation-based mutagenesis and hybridization. For transgenic lines, describe the transformation method, the number of independent lines analyzed and the generation upon which experiments were performed. For gene-edited lines, describe the editor used, the endogenous sequence targeted for editing, the targeting guide RNA sequence (if applicable) and how the editor was applied.*
Authentication	*Describe any authentication procedures for each seed stock used or novel genotype generated. Describe any experiments used to assess the effect of a mutation and, where applicable, how potential secondary effects (e.g. second site T-DNA insertions, mosiacism, off-target gene editing) were examined.*

Flow Cytometry

Plots

Confirm that:

- [x] The axis labels state the marker and fluorochrome used (e.g. CD4-FITC).
- [x] The axis scales are clearly visible. Include numbers along axes only for bottom left plot of group (a 'group' is an analysis of identical markers).
- [x] All plots are contour plots with outliers or pseudocolor plots.
- [x] A numerical value for number of cells or percentage (with statistics) is provided.

Methodology

Sample preparation	HEK293A cells were transfected with human GPR34-encoding plasmid (250 ng), using polyethyleneimine.

Instrument	BD FACSLyric Flow Cytometry System (BD Biosciences, Franklin Lakes, NJ, USA)
Software	FlowJo Software (FlowJo, Ashland, OR, USA)
Cell population abundance	N/A
Gating strategy	Live cells were gated with a forward scatter cutoff of 390 setting again value of 1.7.

☒ Tick this box to confirm that a figure exemplifying the gating strategy is provided in the Supplementary Information.
